# Gut Microbiota and Metabolite Changes in Patients With Ulcerative Colitis and *Clostridioides difficile* Infection

**DOI:** 10.3389/fmicb.2022.802823

**Published:** 2022-05-27

**Authors:** Jian Wan, Yujie Zhang, Wenfang He, Zuhong Tian, Junchao Lin, Zhenzhen Liu, Yani Li, Min Chen, Shuang Han, Jie Liang, Yongquan Shi, Xuan Wang, Lei Zhou, Ying Cao, Jiayun Liu, Kaichun Wu

**Affiliations:** ^1^State Key Laboratory of Cancer Biology, National Clinical Research Center for Digestive Diseases and Xijing Hospital of Digestive Diseases, Fourth Military Medical University, Xi’an, China; ^2^Department of Histology and Embryology, School of Basic Medicine, Xi’an Medical University, Xi’an, China; ^3^Department of Clinical Laboratory, Xijing Hospital, Fourth Military Medical University, Xi’an, China; ^4^Department of Gastroenterology, Honghui Hospital, Xi’an Jiaotong University, Xi’an, China; ^5^Department of Neurology, Xijing Hospital, Fourth Military Medical University, Xi’an, China; ^6^Department of Life Science, Northwest University, Xi’an, China

**Keywords:** ulcerative colitis, gut microbiota, 16S rRNA, metabolites, *Clostridioides difficile* infection

## Abstract

**Background:**

Patients with ulcerative colitis (UC) are at an increased risk of developing *Clostridioides difficile* infection (CDI), which in turn leads to poor outcomes. The gut microbial structure and metabolites in patients with UC and CDI have been scarcely studied. We hypothesized that CDI changes the gut microbiota and metabolites of patients with UC.

**Materials and Methods:**

This study included 89 patients: 30 healthy controls (HC group), 29 with UC alone (UCN group), and 30 with UC and CDI (UCP group). None of the participants has been exposed to antibiotic treatments during the 3 months before stool collection. Stool samples were analyzed using 16S rRNA gene sequencing of the V3–V4 region and gas chromatography tandem time-of-flight mass spectrometry.

**Results:**

The UCN group displayed lower diversity and richness in gut microbiota and a higher relative abundance of the phylum Proteobacteria than the HC group. There were no significant differences between the UCN and UCP groups in the α-diversity indices. The UCP group contained a higher relative abundance of the genera *Clostridium sensu stricto*, *Clostridium* XI, *Aggregatibacter*, and *Haemophilus*, and a lower relative abundance of genera *Clostridium* XIVb and *Citrobacter* than the UCN group. In the UCP group, the increased metabolites included putrescine, maltose, 4-hydroxybenzoic acid, 4-hydroxybutyrate, and aminomalonic acid. Spearman’s correlation analysis revealed that these increased metabolites negatively correlated with *Clostridium* XlVb and positively correlated with the four enriched genera. However, the correlations between hemoglobin and metabolites were contrary to the correlations between erythrocyte sedimentation rate and high-sensitivity C-reactive protein and metabolites.

**Conclusion:**

Our study identified 11 differential genera and 16 perturbed metabolites in patients with UC and CDI compared to those with UC alone. These findings may guide the design of research on potential mechanisms and specific treatments for CDI in patients with UC.

## Introduction

Ulcerative colitis (UC) is a common type of chronic and idiopathic inflammatory bowel disease (IBD) (Ungaro et al., 2017). Patients with UC normally present with mucus and bloody diarrhea (Ungaro et al., 2017). The pathogenesis is multifactorial and results from a dysregulated immune response to environmental factors in genetically susceptible hosts, although the precise pathogenesis has not yet been completely elucidated (Corridoni et al., 2014; Ungaro et al., 2017). The gut microbiome has been implicated in the pathogenesis of UC (Corridoni et al., 2014; Ungaro et al., 2017; Pittayanon et al., 2020). Results from several studies showed that the diversity and richness of gut microbiota in patients with IBD were decreased, including a decrease in the relative abundance of the phylum Firmicutes but an increase in that of phyla Proteobacteria and Actinobacteria (Walker et al., 2011; Lopetuso et al., 2018; Pittayanon et al., 2020).

*Clostridioides difficile* (*C. difficile*) is a Gram-positive, obligate anaerobic bacillus, which can colonize the large intestine. The most common manifestation of *C. difficile* infection (CDI) is diarrhea (Aletaha et al., 2019). Compared to non-CDI patients, patients with CDI showed lower diversity and richness of gut microbiota and an overrepresentation of the members of Bacteroidaceae, Enterococcaceae, Lactobacillaceae, and *Clostridium* XI and XIVa (Perez-Cobas et al., 2014). One study showed that patients with UC had a 4.88 times higher risk of developing a CDI than the non-UC controls (Singh et al., 2017). CDI usually occurs after the gut microbiota has been disturbed, which might be a reason for the high CDI incidence in patients with UC. Furthermore, CDI significantly increases the surgical rate, hospitalization, and cost of treatment for patients with IBD; thus, resulting in a significant increase in IBD-related mortality (Negrón et al., 2016; Law et al., 2017; Rezapour et al., 2018).

Although the gut microbial structure in patients with UC or CDI has been shown to undergo significant change, the gut microbial structure in patients with UC and concomitant CDI has been scarcely studied. Gut microbiota influences the metabolites of the host (Wang et al., 2021). It is imperative to clarify the changes in the gut microbiota and metabolites of patients with UC and CDI to optimize treatment strategies.

We hypothesized that CDI changed the gut microbiota and thereby affected the metabolites of patients with UC. This study aimed to characterize the gut microbiota and metabolites in patients with UC and CDI. Using 16S rRNA gene sequencing and gas chromatography tandem time-of-flight mass spectrometry (GC-TOFMS) metabolomics, we analyzed and compared the gut microbial structure and metabolites from fecal samples of healthy controls and patients with UC with and without CDI. The results might guide the development of specific probiotic treatments and help clinicians prevent CDI development in patients with UC.

## Materials and Methods

### Patients Enrolled and Study Design

The patients in this study were enrolled from an ongoing, prospective cohort study of patients with IBD with or without CDI. From September 2018 until March 2019, we performed 16S rRNA gene sequencing analysis on healthy control patients (HC group), patients with UC alone (UCN group), and patients with UC and CDI (UCP group) visiting the Xijing Hospital in Xi’an, China. All patients from the HC group were free of a recent or chronic illness. None of the participants has been exposed to antibiotic treatments during the 3 months before stool collection. The samples of the patients in the UCP group were taken at the diagnosis of CDI. The UC diagnosis was based on medical history and clinical manifestations combined with endoscopic and histological findings (Magro et al., 2017). The severity of UC was assessed according to the total Mayo score (Rubin et al., 2019). The CDI diagnosis was confirmed by positive results for *C. difficile* culture in the stool samples and positive results for toxin A/B by ELISA. The exclusion criteria were (1) pregnant or breastfeeding and (2) concomitant diagnosis of other infectious diseases, gastrointestinal perforation, or severe heart and liver diseases. The medical history and clinical data of the patients were reviewed. Clinical data included sex, age, disease duration, Mayo score, laboratory test results, and treatments. The stool samples were collected at the Department of Digestive Diseases from Xijing hospital and stored at −80°C for the analyses of microbial communities and untargeted metabolomic detection.

This study was approved by the ethical committee of the Chinese Clinical Trial Registry (ChiECRT20190106). All patients or their legal representatives signed the informed consent form. The trial was registered with https://clinicaltrials.gov/, number NCT04179201. Raw sequencing data have been deposited in the NCBI Sequence Read Archive (SRA) database with accession no. PRJNA753210.

### DNA Isolation and Sequencing

DNA was isolated from samples using the E.Z.N.A.^®^ stool DNA Kit (Omega Bio-Tek, Norcross, GA, United States) according to the manufacturer’s protocols. The V3–V4 region of the bacterial 16S rRNA gene was amplified using PCR. The following primers were used: 341F, 5’-CCTACGGGNGGCWGCAG-3’, and 785R, 5’-GACTACHVGGGTATCTAATCC-3’. PCR reactions were performed in duplicate. The 25-μl reaction mixture contained 10 ng of template DNA, 2.5 μl of 10 × EX Taq Buffer (Takara Bio, Dalian, China), 1 μl of 2.5 mM dNTP (Takara Bio, Dalian, China), 0.5 μl of each primer, 0.1 μl of Ex Taq (Takara Bio, Dalian, China), and PCR-grade water to adjust the volume. PCR reaction cycle consisted of an initial denaturation step at 94°C for 30 s, followed by 20 cycles of denaturation at 94°C for 10 s, annealing at 55°C for 30 s, and extension at 72°C for 30 s, with a final extension at 72°C for 5 min. Next, the amplicons were visualized on 2% agarose gels and purified using the AxyPrep™ Mag PCR Clean-Up Kit (Axygen Biosciences, Union City, CA, United States) according to the manufacturer’s instructions. The quantification process was performed using the Quant-iT™ PicoGreen^®^ dsDNA Assay Kit (Thermo Fischer Scientific, Massachusetts, MA, United States). Purified amplicons were paired-end sequenced on the Illumina Miseq PE300 platform (Illumina, San Diego, CA, United States). The optimized sequences were quality-filtered using Trimmomatic software (Bolger et al., 2014) and merged using Flash software (Magoc and Salzberg, 2011). The filtered sequences were clustered into operational taxonomic units (OTUs) at a 97% similarity level using Uparse software.^1^ The representative sequence of OTUs was compared with the Ribosomal Database Project (RDP) database^2^ by RDP classifier algorithm (Wang et al., 2007) with a confidence threshold of 0.8.

Four α-diversity indices, including observed OTUs, Chao1 index, Simpson index, and Shannon index for evaluating microbial richness and diversity, were calculated using the “vegan” package and visualized by the “ggplot2” package in the R project. The Venn diagrams curve was plotted using the “VennDiagram” package in the R for analyzing group-specific bacterial microbiota. To reflect the β-diversity, we performed the non-metric multidimensional scaling (NMDS) based on Bray–Curtis distance metrics using the “metaMDS” function in the “vegan” package in the R. The differences in the β-diversity of the microbiome were tested by examining measures of Bray–Curtis distance using permutational multivariate analysis of variance (PERMANOVA) using the R vegan package.

The significant differences among the three groups (HC, UCN, and UCP) were analyzed using a Kruskal--Wall’s rank-sum test and the Benjamini--Hochberg correction for multiple hypotheses. Linear discriminant analysis (LDA) effect size (LEfSe)^3^ was further performed to identify the different bacterial taxa between different groups with an LDA score of 3.0 as a cutoff. *P*-values or q-values (false discovery rate adjusted) <0.05 were considered to be significantly different.

### Gas Chromatography Tandem Time-of-Flight Mass Spectrometry-Based Metabolomic Analysis

The untargeted metabolomic analysis was performed by GC-TOFMS. Metabolite extraction, GC-TOF-MS analysis, and data preprocessing and annotation are summarized in the Supplementary Methods. The SIMCA15.0.2 software package (Sartorius Stedim Data Analytics AB, Umea, Sweden) was used for multivariate analysis, including principal component analysis (PCA) and orthogonal projections to latent structures-discriminate analysis (OPLS-DA). PCA was carried out to visualize the distribution and the grouping of the samples. The 95% confidence interval in the PCA score plot was used as the threshold to identify potential outliers in the dataset. The differences among the groups were compared by PERMANOVA using the R vegan package. To visualize group separation and identify significantly changed metabolites, supervised OPLS-DA was applied. The variable importance for the projection (VIP) parameter was calculated by the OPLS-DA model. It summarizes the contribution of each variable to the model. The metabolites with VIP > 1 and *p* < 0.05 (Student’s *t*-test) were considered as significantly changed metabolites.

### Analysis of Laboratory Tests

Blood samples were collected for analysis by laboratory tests using automated equipment and standard methods. Hemoglobin, erythrocyte sedimentation rate (ESR), and high-sensitivity C-reactive protein (hs-CRP) were examined within 1 week before the stool sample was taken.

### Statistical Analyses

Differences in the baseline patient characteristics were analyzed using SPSS 19.0 (IBM, Armonk, NY, United States). The version of R software used in our study was 3.6.1 (the R Project for Statistical Computing^4^). Quantitative variables were summarized as the median and interquartile range (IQR). The Student’s *t*-test or Wilcoxon rank-sum test was used to compare mean values, as appropriate. Categorical variables were expressed as frequencies and percentages, and the χ^2^ test or Fisher’s exact test was used to distinguish them when necessary. The correlations between the gut microbiota and metabolites were assessed with Spearman’s correlation test using R. The tests were two-tailed, and *p*-values < 0.05 were considered to be statistically significant.

## Results

### Participant Characteristics

The 89 patients included in our study were divided into the HC (30 subjects), UCN (29 subjects), and UCP (30 subjects) groups. The patients’ characteristics are presented in Table 1. The three groups were similar in sex distribution and age. The UCN and UCP groups were similar in the following variables: disease duration, Mayo score, and the treatment for UC 6 months before sample collection, which included the use of 5-ASA, corticosteroids, azathioprine, and biologics (all *p* > 0.05). Almost all patients with UC (58/59, 98.3%) have been treated with 5-ASA. The number of patients treated with corticosteroid, immunosuppressor, and biologics 6 months before the study was 21 (35.6%), 3 (5.1%), and 5 (8.5%), respectively.

**TABLE 1:** Characteristics of patients.

Characteristics	HC (*n* = 30)	UCN (*n* = 29)	UCP (*n* = 30)	*p*-value
Sex				0.848
Female	10 (33.3)	10 (34.5)	12 (40.0)	
Male	20 (66.7)	19 (65.5)	18 (60.0)	
Age (>45 years)	22 (73.3)	17 (58.6)	14 (46.7)	0.108
Disease duration, year, median, IQR	NA	2.0 (0.5–6.5)	2.0 (1.0–6.3)	0.818*
UC activity				0.490*
Clinical remission	NA	1	0	
Mild	NA	5	2	
Moderate	NA	16	20	
Severe	NA	7	8	
5-ASA	NA	29 (100.0)	29 (96.7)	1.000*
Corticosteroids	NA	9 (31.0)	12 (40.0)	0.472*
Azathioprine	NA	1 (3.4)	2 (6.7)	1.000*
Biologics	NA	1 (3.4)	4 (13.3)	0.353*

*p-value of the UCN vs. UCP groups. 5-ASA, 5-aminosalicylic acid; IQR, interquartile range; HC, healthy control group; NA, not available; UCN, ulcerative colitis with negative Clostridioides difficile test results; UCP, ulcerative colitis with positive C. difficile infection.

### 16S rRNA Gene Sequencing and Diversity Analysis

**FIGURE 1:**
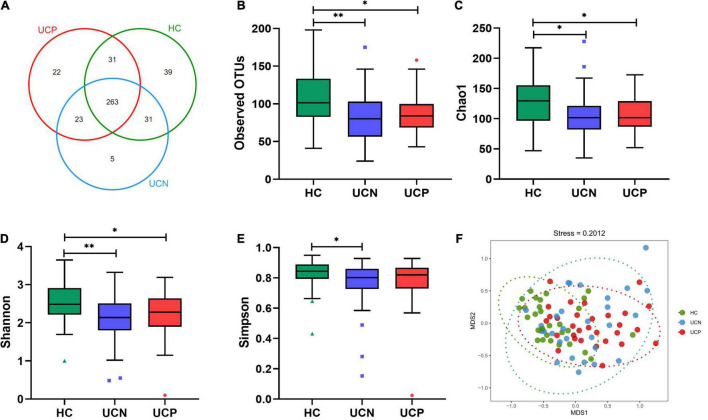
Comparison of gut microbial structure among the HC, UCN, and UCP groups. **(A)** The Venn diagram shows the overlapping OTUs from the gut microbiomes of the three groups. **(B–E)** Alpha-diversity indices (the box plots of observed OTUs, Chao1, and the Shannon and the Simpson indices) among the three groups. *P < 0.05, ^**^P < 0.01. **(F)** Beta-diversity index (plots of NMDS) among the three groups. HC, healthy control group; OTUs, operational taxonomic units; UCN, ulcerative colitis with negative *Clostridioides difficile* test results; UCP, ulcerative colitis with positive *Clostridioides difficile* infection.

The 16S rRNA gene sequencing was used to assess the changes in gut microbiota between the HC and UCN groups and between the UCN and UCP groups. A total of 4,526,326 high-quality raw reads were generated using the Illumina MiSeq platform. We obtained 3,291,816 clean reads (37,340.0, 36,736.6, and 36,875.1 average reads for HC, UCN, and UCP groups, respectively) after filtering. A total of 414 OTUs were identified at a 97% similarity level. These 414 OTUs belonged to 147 different genera in 11 different phyla. Of these OTUs, 263 were shared among the three groups, and 39, 5, and 22 OTUs were specific to the HC, UCN, and UCP groups, respectively (Figure 1A). The four α-diversity indices were higher for the HC group than for the UCN group (*p*-values < 0.05). This indicates that the UCN group displayed less diversity and richness in gut microbiota than the HC group. There were no significant differences between the UCN and UCP groups in the four α-diversity indices (all *p*-values > 0.05). These results indicate that the UCN and UCP groups displayed similar diversity and richness in their gut microbiota. The observed OTUs and Chao1, Simpson, and Shannon indices are shown in Figures 1B–E. For β-diversity index, the NMDS of Bray–Curtis with a stress value of 0.20 is shown in Figure 1F. The PERMANOVA revealed significant differences in gut microbial community structure between the HC and UCN groups (*P* = 0.001). However, we found no difference in the gut microbiota composition between the UCN and UCP groups (PERMANOVA, *P* = 0.147).

### Changes in Gut Microbiota Between the HC and UCN Groups

**FIGURE 2:**
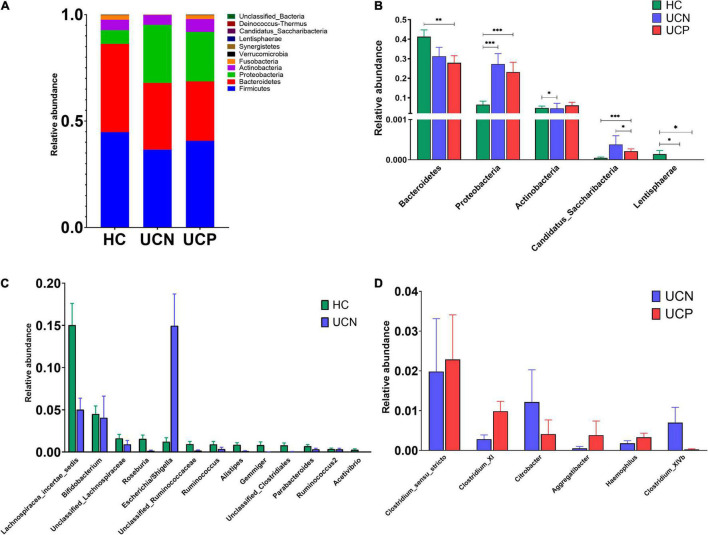
**(A)** The structure of the gut microbiota at the phylum level among the three groups. **(B)** The significantly different taxa at the phylum level among the three groups. *P < 0.05, ^**^P < 0.01, ^***^P < 0.001. **(C)** The significantly different taxa at the genus level between the HC and UCN groups. **(D)** The significantly different taxa at the genus level between the UCN and UCP groups. HC, healthy control group; UCN, ulcerative colitis without negative *Clostridioides difficile* test results; UCP, ulcerative colitis with positive *Clostridioides difficile* infection.

At the phylum level, 11 phyla were detected in the three groups. The most abundant phylum was Firmicutes, followed by Bacteroidetes, Proteobacteria, Actinobacteria, and Fusobacteria. The sum of the relative abundance of these five phyla was more than 99% in each group (Figure 2A). Compared to the HC group, the relative abundance of the phylum Proteobacteria in the UCN group was markedly increased (6.55% in HC, 27.26% in UCN, q < 0.001), whereas the relative abundance of the phylum Actinobacteria (5.82% in HC, 4.59% in UCN, q = 0.011) and Lentisphaerae (0.01% in HC, 0% in UCN, q = 0.042) was decreased (Figure 2B). Nevertheless, there was no significant difference in the relative abundance of phylum Firmicutes (44.83% in HC, 36.63% in UCN, q = 0.124), Bacteroidetes (41.35% in HC, 31.26% in UCN, q = 0.117), and Fusobacteria (2.27% in HC, 0.14% in UCN, q = 0.408) between the HC and UCN groups.

A total of 147 genera were identified in the three groups. There were 35 genera (phyla are indicated in parentheses) with significant changes in the relative abundance between the HC and UCN groups. *Escherichia*/*Shigella* (Proteobacteria) was enriched in the UCN group compared to the HC group. Furthermore, the relative abundance of *Lachnospiracea incertae sedis* (Firmicutes), *Bifidobacterium* (Actinobacteria), unclassified *Lachnospiraceae* (Firmicutes), *Roseburia* (Firmicutes), unclassified *Ruminococcaceae* (Firmicutes), *Ruminococcus* (Firmicutes), *Alistipes* (Bacteroidetes), *Gemmiger* (Firmicutes), unclassified *Clostridiales* (Firmicutes), *Parabacteroides* (Bacteroidetes), *Ruminococcus 2* (Firmicutes), and *Acetivibrio* (Firmicutes) was decreased in the UCN group (Figure 2C). The relative abundance of the other 22 significantly differential genera was low (less than 0.2%).

**FIGURE 3:**
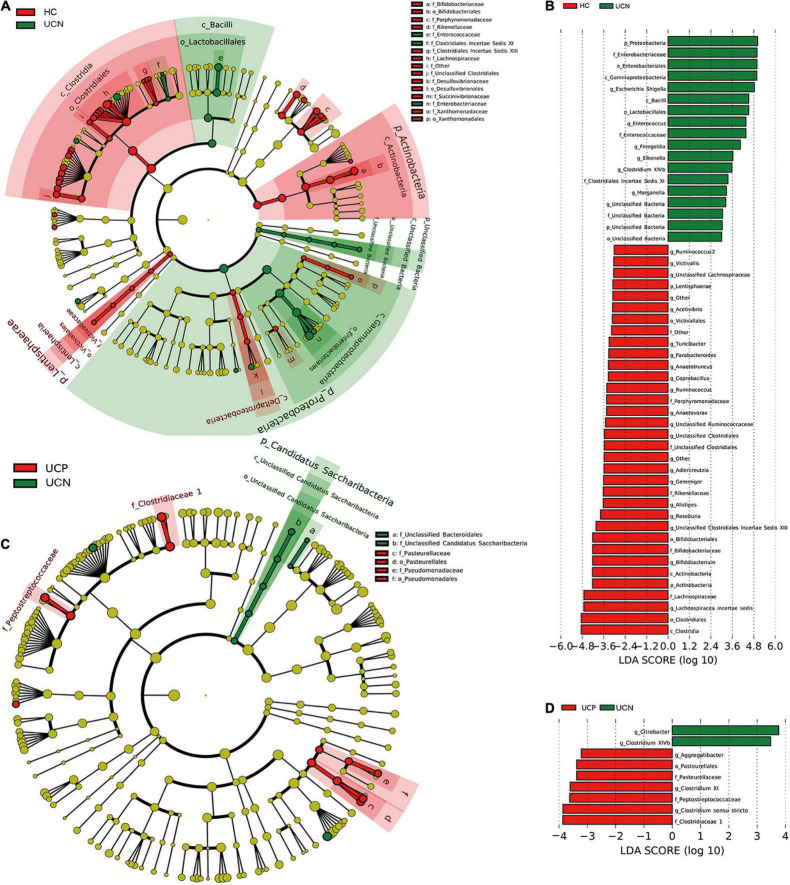
Differential relative abundance of bacterial taxa among the three groups. **(A)** A cladogram made by LEfSe demonstrates different bacterial taxa between the HC and UCN groups. **(B)** LDA score of enriched bacterial taxa between the HC and UCN groups. Only taxa meeting an LDA > 3 are shown. **(C)** A cladogram made by LEfSe demonstrates different bacterial taxa between the UCN and UCP groups. **(D)** LDA score of enriched bacterial taxa between the UCN and UCP groups. Only taxa meeting an LDA > 3 are shown. HC, healthy control group; UCN, ulcerative colitis with negative *Clostridioides difficile* test results; UCP, ulcerative colitis with positive *Clostridioides difficile* infection.

We also performed LEfSe analysis to test different bacterial taxa between the groups. LEfSe analysis is a high-dimensional biomarker discovery algorithm, which uses LDA to estimate the effective size of the difference in each distinct taxon between different groups. A cladogram was used to demonstrate the relationship between the HC and UCN groups (Figure 3A). LEfSe analysis identified 52 different taxa between the HC and UCN groups with an LDA ≥ 3 (Figure 3B), including 4 phyla, 4 classes, 6 orders, 10 families, and 28 genera.

### Changes in Gut Microbiota Between the UCN and UCP Groups

The composition of gut microbiota at the phylum level was almost identical in the UCN and UCP groups. There was a significant difference in phylum Candidatus Saccharibacteria (0.04% in UCN, 0.02% in UCP, q = 0.029) (Figure 2B), whereas no significant differences between the UCN and UCP groups in the relative abundance of the following phyla were identified: Firmicutes (36.63 vs. 40.69%, q = 0.323), Bacteroidetes (31.26 vs. 27.92%, q = 0.552), Proteobacteria (27.26% vs. 23.20%, q = 0.816), Actinobacteria (4.59 vs. 6.12%, q = 0.065), and Fusobacteria (0.14 vs. 1.80%, q = 0.558).

There were 11 genera (phyla are indicated in parentheses) that showed significantly different relative abundance levels in the UCN group compared to the UCP group. Compared to the UCN group, *Clostridium sensu stricto* (Firmicutes), *Clostridium* XI (Firmicutes), *Aggregatibacter* (Proteobacteria), and *Haemophilus* (Proteobacteria) were enriched in the UCP group. Conversely, the relative abundance levels of *Clostridium* XIVb (Firmicutes) and *Citrobacter* (Proteobacteria) were decreased in the UCP group when compared to those in the UCN group (Figure 2D). The relative abundance of the other five significantly different genera was low (less than 0.2%). The relative abundance of *Turicibacter* (Firmicutes), *Pseudomonas* (Proteobacteria), and *Neisseria* (Proteobacteria) was higher in the UCP group. Concurrently, the relative abundance of *Saccharibacteria genera incertaesedis* (Candidatus Saccharibacteria) and *Solobacterium* (Firmicutes) was lower in the UCP group.

A cladogram was used to demonstrate the relationship between the UCN and UCP groups (Figure 3C). LEfSe analysis identified nine different taxa between the UCN and UCP groups with an LDA ≥ 3 (Figure 3D), including one order, three families, and five genera.

### Metabolome Analysis

**FIGURE 4:**
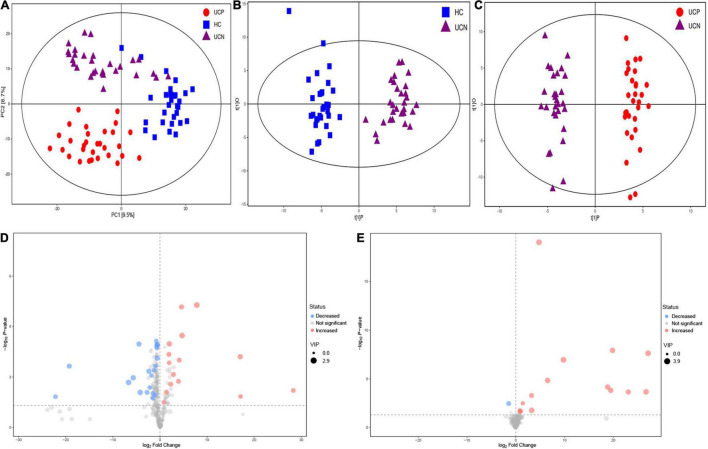
**(A)** PCA score plot of metabolite profile among the three groups. **(B)** OPLS-DA score plots of samples from the HC and UCN groups. **(C)** OPLS-DA score plots of samples from the UCN and UCP groups. **(D)** Volcano plots of differential metabolites between the UCN and HC groups, where red dots represent increased metabolites and blue dots represent decreased metabolites. **(E)** Volcano plots of differential metabolites between the UCP and UCN groups, where red dots represent increased metabolites and blue dots represent decreased metabolites. HC, healthy control group; UCN, ulcerative colitis with negative *Clostridioides difficile* test results; UCP, ulcerative colitis with positive *Clostridioides difficile* infection.

In our study, 407 peaks were detected, and 311 metabolites were left after relative standard deviation de-noising. Multivariate analysis methods, including PCA and OPLS-DA, were used to identify metabolite changes. PCA analysis was performed to assess the profile differences among the three groups (Figure 4A). The results of the PCA analysis showed a clear separation between the groups, which suggested that the three groups had different metabolic profiles (PERMANOVA, *P* = 0.001). At the same time, the score plot of OPLS-DA revealed a distinction between the HC and UCN groups (R_2_Y = 0.934, Q_2_ = 0.771) (Figure 4B). A distinction was also found between the UCN and UCP groups (R_2_Y = 0.974, Q_2_ = 0.855) (Figure 4C). These results indicated a significant difference in the metabolite profile among the three groups.

The VIP parameter > 1.0 and *p*-value < 0.05 (two-tailed Student’s *t*-test) were used to identify significant differences in metabolites between the groups. As shown in Figure 4D, a total of 34 differential metabolites between the UCN and HC groups were identified, including 15 elevated and 19 decreased metabolites in the UCN group (Supplementary Table 1). As shown in Figure 4E, a total of 16 differential metabolites between the UCP and UCN groups were identified, including 15 elevated and one decreased metabolite in the UCP group (Table 2).

**TABLE 2:** Significantly different metabolites between the UCP and UCN groups.

Metabolites	VIP	Fold change	*P*-value	Enrich
Putrescine 2	3.4066	1.11E+08	0.00021613	UCP
Lactic acid	2.2508	9.41	0.000511849	UCP
Mannose 1	3.5791	1.48E+08	2.3319E-08	UCP
Lyxose 1	3.4330	9.66	0.01719414	UCP
Maltose	2.9975	7.37E+05	0.000151701	UCP
3-Phenyllactic acid	1.2598	1.85	0.025133089	UCP
Aminomalonic acid	3.2537	92.33	1.45944E-05	UCP
Sophorose 2	2.5848	8.96E+06	0.000226705	UCP
4-Hydroxybutyrate	3.9304	883.39	1.105E-07	UCP
3-Hydroxynorvaline 2	1.2537	1.36	0.030209161	UCP
Hexadecane	2.2264	0.37	0.003416379	UCN
5-Dihydrocortisol 1	3.0530	4.70E+05	6.98172E-05	UCP
Uridine 2	1.8784	2.02	0.020053092	UCP
Coniferyl alcohol	1.3666	2.73	0.003222153	UCP
4-Hydroxybenzoic acid	3.1645	9.43E+05	1.18732E-08	UCP
2-Deoxyuridine	3.9173	27.27	9.31737E-20	UCP

*UCN, ulcerative colitis with negative Clostridioides difficile test results; UCP, ulcerative colitis with positive Clostridium difficile infection; VIP, variable importance for projection.*

### Correlation Among Gut Metabolites, Microbiota, and Laboratory Tests

**FIGURE 5:**
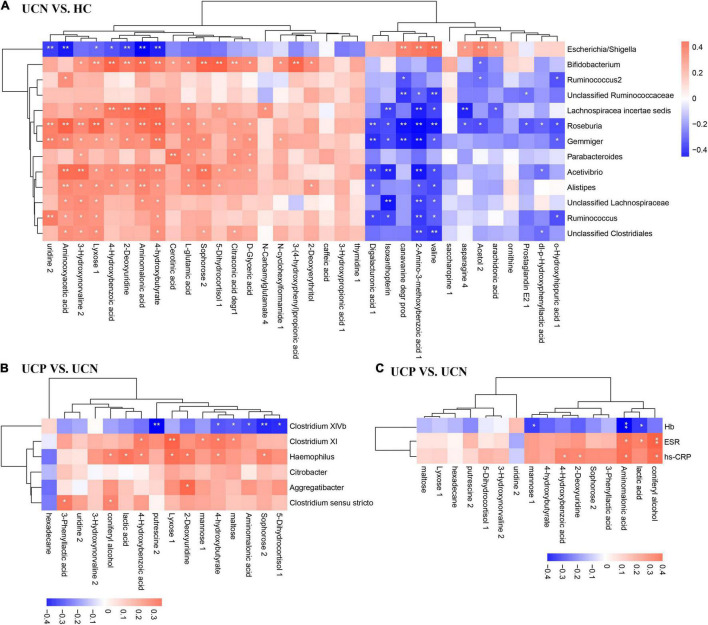
Correlation analysis of differential gut microbiota genera (relative abundance > 0.2%) and differential metabolites between the HC and UCN groups **(A)**, and between the UCN and UCP groups **(B)**. **(C)** Correlation analysis of differential metabolites and laboratory tests between the UCN and UCP groups. *p < 0.05; **p < 0.01; HC, healthy control group; UCN, ulcerative colitis with negative *Clostridioides difficile* test results; UCP, ulcerative colitis with positive *Clostridioides difficile* infection.

Spearman’s correlation coefficient was calculated for the differential metabolites and differential genus-level bacterial taxa. Figure 5A showed the correlations between the 13 differential gut microbiota (relative abundance > 0.2%) and 34 differential metabolites between the HC and UCN groups. The genus *Escherichia/Shigella*, enriched in the UCN group, presented positive correlations with six metabolites, including valine, 2-amino-3-methoxybenzoic acid 1, canavanine degr prod (named canavanine in KEGG compound database), acetol 2, arachidonic acid, and asparagine 4, which were increased in the UCN group. The genus *Escherichia/Shigella* also negatively correlated with seven metabolites, including aminomalonic acid, aminooxyacetic acid, 4-hydroxybutyrate, uridine 2, 2-deoxyuridine, 4-hydroxybenzoic acid, and lyxose 1, which were decreased in the UCN group. However, the correlation between the 12 decreased genera and the increased/decreased metabolites was completely contrary to the correlation between genus *Escherichia/Shigella* and the increased/decreased metabolites.

Figure 5B showed the correlation between the six differential gut microbiota (relative abundance > 0.2%) and 16 differential metabolites between the UCN and UCP groups. The genus *Clostridium* XIVb (phylum Firmicutes), decreased in the UCP group, showed a negative correlation with six metabolites, including 4-hydroxybutyrate, 5-dihydrocortisol 1, aminomalonic acid, maltose, putrescine 2, and sophorose 2. The correlation between the other four enriched genera in the UCP group and the increased/decreased metabolites was completely contrary to the correlation between genus *Clostridium* XIVb and the increased/decreased metabolites.

Figure 5C shows the correlation between three laboratory tests (Hb, ESR, and hs-CRP) and 16 differential metabolites between the UCN and UCP groups. The metabolites that were increased in the UCP group, which included coniferyl alcohol, aminomalonic acid, lactic acid, 2-deoxyuridine, and 4-hydroxybenzonic acid, presented a positive correlation with ESR and hs-CRP. At the same time, the correlation between Hb and these metabolites was completely contrary to the correlation between ESR and hs-CRP and these metabolites.

## Discussion

Ulcerative colitis patients had a 4.88-fold increase in the risk of CDI than individuals without UC (Singh et al., 2017). CDI significantly increases the surgical rate, hospitalization, cost of treatment for patients, and UC-related mortality (Negrón et al., 2016; Law et al., 2017; Rezapour et al., 2018). The study of gut microbiota and metabolite changes in the patients with UC after CDI might guide the development of specific treatments for CDI and help clinicians prevent CDI development in patients with UC. This was the most extensive study presenting specific gut microbiota and metabolite differences between patients with UC with and without CDI to the best of our knowledge. Herein, we revealed that gut microbiota structure and metabolite profile were significantly different between healthy controls and patients with UC and between patients with UC with and without CDI using 16S r RNA sequencing and GC-TOFMS analysis.

Numerous previous studies have demonstrated different results regarding the changes in the gut microbiota in patients with UC (Lopetuso et al., 2018; Pittayanon et al., 2020). At the phylum level, the most significant changes have been observed in the population of Proteobacteria in children with severe UC (Michail et al., 2012). Furthermore, the phylum Proteobacteria was increased in the patients of both UC and Crohn’s disease (CD) in a large-sample, case-control study (Imhann et al., 2018). In line with previous findings, we showed that the most dramatically increased phylum was Proteobacteria (Michail et al., 2012; Imhann et al., 2018). Lower diversity and richness in the gut microbiota and decreased relative abundance of family Lachnospiraceae were observed in UC (Schirmer et al., 2018). In this study, the most decreased genera belonged to the family Lachnospiraceae, including *Lachnospiracea incertae sedis*, unclassified *Lachnospiraceae*, and *Roseburia*. The genera *Roseburia* and *Ruminococcus* were shown to be reduced in the patients with UC (Rodriguez et al., 2020), which was consistent with our findings. *Escherichia coli* was the most common bacteria found to be increased in the patients with UC (Pittayanon et al., 2020). This was consistent with our results associated with the genus *Escherichia*/*Shigella*, which cannot be distinguished by 16S rRNA gene sequences.

Patients with UC are at increased risk for CDI, and patients with UC and CDI are more likely to have poor outcomes than patients with UC alone (Negrón et al., 2016; Law et al., 2017; Rezapour et al., 2018). Results from various studies have shown that significant changes occur in the microbiome of patients with CDI when compared to that of healthy controls (Gu et al., 2016; Lamendella et al., 2018). Patients with CDI had a lower relative abundance of Ruminococcaceae and Lachnospiraceae than control patients without CDI (Lamendella et al., 2018). Furthermore, patients with CDI exhibited lower diversity and different microbial compositions than healthy controls (Ross et al., 2016). Interestingly, the UCN and UCP groups had similar α-diversity in our study. This result was similar to the results of Sokol et al. (2018), which demonstrated that there was no significant difference in IBD flare between patients with and without CDI. This might indicate that CDI did not change the relative abundance of most of the gut microbiota but rather resulted in a change in specific important pathogens, such as *Clostridium sensu stricto*, *Clostridium* XI, and *Clostridium* XIVb. Such changes could lead to severe consequences. Our study aimed to find the differences in changes in gut microbiome between patients with UC and CDI. This might be of value for the development of drugs for treating CDI in patients with UC.

Although several studies have focused on the changes in gut microbiota in patients with UC or CDI, the gut microbial structure in patients with UC and CDI has rarely been studied (Rodriguez et al., 2020). Patients with IBD and CDI had higher levels of *Ruminococcus gnavus* and *Enterococcus* and lower levels of *Blautia* and *Dorea* than patients with IBD without CDI (Sokol et al., 2018). In Sokol et al.’s study, patients with both UC and CD were included. However, only two patients exhibited UC and CDI (Sokol et al., 2018). As previous studies have shown, the changes in the gut microbiota of patients with UC are different than those of patients with CD (Imhann et al., 2018). In our study, the most increased genera were *Clostridium sensu stricto* and *Clostridium* XI, whereas the primarily decreased genera were *Clostridium* XIVb and Citrobacter. The family Clostridiaceae 1 was significantly increased in patients with CDI (Gu et al., 2016), a result that was similar to our findings, which indicated higher relative abundance in the UCP group than in the UCN group. In our study, this result was mainly due to a significant increase in the genus *Clostridium sensu stricto*. *Clostridium sensu stricto*, considered as a harmful bacteria, could also have adverse effects on the intestinal tract (Zhou et al., 2015). *Clostridium* XI (*C. difficile* belongs to this genus) is a well-known proinflammatory and colitis-inducing bacterium (Perez-Cobas et al., 2014; Aletaha et al., 2019). The cladogram shows that *Clostridium* XI belonged to the family Peptostreptococcaceae. The increased relative abundance of genus *Clostridium* XI and family Peptostreptococcaceae might be attributed to the presence of *C. difficile* in the UCP group. *Clostridium* XIVb, which was decreased in the UCP group, was associated with the integrity of the intestinal barrier and the production of short-chain fatty acids (SCFAs) (Bajaj et al., 2019). This suggested that *Clostridium* XIVb might play an important role in CDI development in patients with UC, and depletion of *Clostridium* XIVb might contribute to CDI in patients with UC.

As we know, changes in the gut microbial community may lead to alterations in the metabolite profile. The disordered metabolites may enter the host, modulate the intestinal epithelial cells, and finally affect the balance between pro- and anti-inflammatory mechanisms (Arpaia et al., 2013). Therefore, we examined the changes in metabolites and analyzed the correlation between the differential metabolites, gut microbiota, and inflammation-related laboratory tests. In our study, we detected a total of 16 significant metabolites in the UCP groups compared to the UCN groups, 15 of which were increased and one was decreased. Among these metabolites, putrescine 2 (named putrescine in the KEGG compound database) belonged to polyamines (a kind of amines) and was the most dramatically increased metabolite. A significantly negative correlation between putrescine 2 and the relative abundance of *Clostridium* XIVb was detected. Increased polyamine synthesis was a consequence of inflammation (Babbar et al., 2007). Putrescine might have a strong role in the regeneration of intestinal epithelium (Forget et al., 1997). The presence of CDI may lead to an enhanced inflammatory response in IBD patients (Monaghan et al., 2015). The reason for the higher level of putrescine may be due to more severe inflammation and destruction of intestinal epithelium in patients with UC and CDI. Maltose is a common nutritional disaccharide generated in the gut during starch digestion (Qi and Tester, 2020). Disaccharides could not be absorbed in the gut but could be converted by the digestive enzymes to monosaccharides which could be absorbed (Qi and Tester, 2020). We demonstrated that the UCP group had a higher level of maltose. Maltose is a byproduct formed during the metabolism of starch and sucrose and showed a positive correlation with *Clostridium* XI and a negative correlation with *Clostridium* XIVb. Water retention in the intestine due to the osmotic pressure of unabsorbed disaccharides may be another important causal mechanism for diarrhea in UC patients with CDI. Although 4-hydroxybenzoic acid could come from diets rich in plant-based foods, evidence suggests that it could be produced through microbial fermentation of aromatic amino acids in the colon (Russell et al., 2013). The change in 4-hydroxybenzoic acid level possibly indicates alterations in the gut microbiota after CDI in patients with UC. In our study, 4-hydroxybenzoic acid was increased in the UCP group and positively correlated with hs-CRP, the genera *Haemophilus*, and *Clostridium* XI. Increased *Escherichia* spp. accompanied by increased 4-hydroxybutyrate appeared to reduce butyrate production (Chiu et al., 2019). Butyrate, a type of SCFA, plays a trophic role as a nutrient for colonocytes and enhances the repair of the injured gut epithelium in IBD (Oliver et al., 2021). This suggests that 4-hydroxybutyrate might exhibit an adverse effect on the tissue repair of epithelial cells. 4-Hydroxybutyrate is a kind of hydroxy fatty acid and could be produced by *Pseudomonas putida* (belongs to the genus *Pseudomonas*) (Zhang et al., 2009). In our study, 4-hydroxybutyrate, which was increased in the UCP group, positively correlated with *Clostridium* XI, *Haemophilus*, and *Pseudomonas* (data not shown in the article) and negatively correlated with *Clostridium* XIVb. Several studies demonstrated that the change in aminomalonic acid level was associated with neuropsychiatric disorders, hepatocellular carcinoma, and aneurysm (Xue et al., 2008; Ruperez et al., 2012; Chen et al., 2018), indicating that aminomalonic acid is an essential indicator for diseases and toxicities. It has been reported that an increase in aminomalonic acid level may be related to iron metabolism (Ju et al., 2013). This suggested that anemia in UCP patients is not only associated with excessive blood loss but also with abnormal iron metabolism. In the present study, aminomalonic acid was negatively correlated with *Clostridium* XIVb and Hb, and positively correlated with ESR and CRP.

There is ample evidence that the gut microbiota contributes to the pathogenesis of CDI (Bassis et al., 2014; Zhang et al., 2015). Changes in the gut microbial community could lead to changes in the levels of different metabolites. Therefore, the characterization of gut microbiota and metabolites associated with CDI in patients with UC and elucidation of the exact functional role of the intestinal microbiota and metabolites in the disease pathogenesis must be further investigated. Our study may be of value by promoting the design of specific treatment for CDI in patients with UC.

Although this is the most extensive study presenting the differences in gut microbiota and metabolites between patients with UC with and without CDI, it has various limitations. First, the study sample is small and lacks a validation cohort. Second, 16S sequencing analysis lacks the necessary resolution to classify down to the species level or occasionally, even at the genus level accurately. Third, diet affects the gut microbiota and metabolite profile; therefore, further studies should be performed in patients on the same diets (Wu et al., 2011).

## Conclusion

This study demonstrated significant differences in the gut microbiota and related metabolites between the healthy control patients and patients with UC alone, and between patients with UC alone and patients with UC and CDI. Compared to the HC group, the UCN group displayed less diversity and richness in gut microbiota and a higher relative abundance of the phylum Proteobacteria. Alternatively, there were no significant differences between the UCN and UCP groups in the α-diversity indices. The microbiome of the UCP patients contained a higher relative abundance of the genera *Clostridium sensu stricto*, *Clostridium* XI, *Aggregatibacter*, and *Haemophilus*, and a lower relative abundance of the genera *Clostridium* XIVb and *Citrobacter* when compared to that of patients from the UCN group. The increased metabolites in the UCP group, including putrescine, maltose, 4-hydroxybenzoic acid, 4-hydroxybutyrate, and aminomalonic acid, were negatively correlated with *Clostridium* XIVb and positively correlated with the four enriched genera. The correlation of hemoglobin with metabolites was contrary to that shown by both erythrocyte sedimentation rate and high-sensitivity C-reactive protein. Our study may be of value in promoting prevention and designing specific treatment for CDI in patients with UC. Future studies should focus on whether these significantly changed species could be used as a marker for predicting the development of CDI. Also, whether the significantly decreased species in the UCP group could be used to prevent and treat CDI in the patients with UC is our direction of future research.

## Data Availability Statement

The datasets presented in this study can be found in online repositories. The names of the repository/repositories and accession number(s) can be found below: https://www.ncbi.nlm.nih.gov/, PRJNA753210.

## Ethics Statement

The studies involving human participants were reviewed and approved by the Ethical Committee of the Chinese Clinical Trial Registry (ChiECRT20190106). The patients/participants provided their written informed consent to participate in this study.

## Author Contributions

KW designed and supervised the study. JW, YZ, and WH collected the data. ZT, YC, JLin, WH, LZ, and JLiu provided technical help. MC, ZL, YL, SH, JLia, and YS recruited the patients. JW, XW, and YZ performed the analysis and interpreted the data. JW and YZ wrote the manuscript. YC, JLiu, and KW revised the manuscript. All authors state that the manuscript, including related data, figures, and tables has not been previously published and that the manuscript is not under consideration elsewhere and approved the final version of the manuscript.

## Conflict of Interest

The authors declare that the research was conducted in the absence of any commercial or financial relationships that could be construed as a potential conflict of interest.

## Publisher’s Note

All claims expressed in this article are solely those of the authors and do not necessarily represent those of their affiliated organizations, or those of the publisher, the editors and the reviewers. Any product that may be evaluated in this article, or claim that may be made by its manufacturer, is not guaranteed or endorsed by the publisher.

## Footnotes

1
http://www.drive5.com/usearch/manual/uparseotu_algo.html

2
http://rdp.cme.msu.edu/misc/resources.jsp

3
http://huttenhower.sph.harvard.edu/galaxy

4
http://www.R-project.org
