# Supplementary material for: Gut Microbiota and Metabolite Changes in Patients With Ulcerative Colitis and Clostridioides difficile Infection
Source: Front Microbiol. 2022 May 27;13:802823. doi: 10.3389/fmicb.2022.802823 (PMC9231613; doi:10.3389/fmicb.2022.802823)
Supplement: Supplementary file 1 [file Table_1.docx]

**Supplementary Methods:**

**Metabolites Extraction:**

Approximately 50 mg sample was transferred into a 2 mL tube, and 500 μL pre-cold extraction mixture (methanol/chloroform (v:v) =3:1) with 10 μL internal standard (L-2-Chlorophenylalanine, 1 mg/mL stock) were added.

Samples were vortexed for 30 s and homogenized with ball mill for 4 min at 35 Hz, followed by ultrasonication for 5 min in ice water. After centrifugation at 4 ℃ for 15 min at 10000 rpm, 200 μL supernatant was transferred to a fresh tube. To prepare the QC (Quality control) sample, 60 μL of each sample was taken out and combined together.

After evaporation in a vacuum concentrator, 60 μL of Methoxyamination hydrochloride (20 mg/mL in pyridine) was added and then incubated at 80 ℃ for 30 min, then derivatized by 80 μL of BSTFA regent (1% TMCS, v/v) at 70 ℃ for 1.5 h. Gradually cooling samples to room temperature, 5 μL of FAMEs (in chloroform) was added to the QC sample. All samples were then analyzed by gas chromatography coupled with a time-of-flight mass spectrometer (GC-TOF-MS).

**GC-TOF-MS Analysis：**

GC-TOF-MS analysis was performed using an Agilent 7890 gas chromatography coupled with a time-of-flight mass spectrometer. The system utilized a DB-5MS capillary column. 1 μL aliquot of sample was injected in splitless mode. Helium was used as the carrier gas, the front inlet purge flow was 3 mL min−1, and the gas flow rate through the column was 1 mL min−1. The initial temperature was kept at 50 °C for 1 min, then raised to 310 °C at a rate of 10 °C min−1, then kept for 8 min at 310 °C. The injection, transfer line, and ion source temperatures were 280, 280and 250 °C, respectively. The energy was -70 eV in electron impact mode. The mass spectrometry data were acquired in full-scan mode with the m/z range of 50-500 at a rate of 12.5 spectra per second after a solvent delay of 6.33 min.

**Data preprocessing and annotation：**

Raw data analysis, including peak extraction, baseline adjustment, deconvolution, alignment, and integration, was finished with Chroma TOF (V 4.3x, LECO) software [1] and the LECO-Fiehn Rtx5 database was used for metabolite identification by matching the mass spectrum and retention index. Finally, the peaks detected in less than half of QC samples or RSD>30% in QC samples were removed [2].

**References**

[1]. Kind T, Wohlgemuth G, Lee DY, et al. FiehnLib: mass spectral and retention index libraries for metabolomics based on quadrupole and time-of-light gas chromatography/mass spectrometry. Anal Chem 2009; 81(24): 10038-48.

[2]. Dunn WB, Broadhurst D, Begley P, et al. Procedures for large-scale metabolic profiling of serum and plasma using gas chromatography and liquid chromatography coupled to mass spectrometry. Nat Protoc 2011; 6(7): 1060-83.

| Supplementary Table 1. Significant different metabolites between the UCN and HC groups. | | | | |
| --- | --- | --- | --- | --- |
| Metabolites | VIP | Fold change | P-value | Enrich |
| valine | 2.2714 | 3.97 | 1.07261E-05 | UCN |
| Lyxose 1 | 2.3095 | 0.06 | 0.008329259 | HC |
| Aminomalonic acid | 2.2984 | 0.02 | 0.001121058 | HC |
| arachidonic acid | 1.2828 | 3.91 | 0.00014436 | UCN |
| 3-(4-hydroxyphenyl)propionic acid | 1.1561 | 0.36 | 0.018712451 | HC |
| 3-Hydroxypropionic acid 1 | 1.2036 | 0.41 | 0.000401015 | HC |
| ornithine | 1.6019 | 15.21 | 0.001821476 | UCN |
| thymidine 1 | 1.0267 | 0.49 | 0.011712611 | HC |
| dl-p-Hydroxyphenyllactic acid | 1.5115 | 2.59 | 0.007980826 | UCN |
| asparagine 4 | 1.6566 | 17.16 | 9.88735E-05 | UCN |
| D-Glyceric acid | 2.0500 | 0.67 | 1.23141E-05 | HC |
| Sophorose 2 | 1.3720 | 2.19E-07 | 0.01498983 | HC |
| 2-Deoxyerythritol | 1.3306 | 0.35 | 0.009525503 | HC |
| Digalacturonic acid 1 | 2.4968 | 25.86 | 3.42811E-06 | UCN |
| 4-hydroxybutyrate | 2.4572 | 0.01 | 0.002113851 | HC |
| Cerotinic acid | 1.2864 | 0.22 | 0.004216366 | HC |
| L-glutamic acid | 1.5992 | 0.15 | 0.00823683 | HC |
| N-Carbamylglutamate 4 | 1.2063 | 1.42E+05 | 0.014486225 | UCN |
| Prostaglandin E2 1 | 1.2831 | 1.44 | 0.049612077 | UCN |
| 3-Hydroxynorvaline 2 | 1.1152 | 0.49 | 0.000222018 | HC |
| 2-Amino-3-methoxybenzoic acid 1 | 2.7513 | 225.52 | 5.23353E-08 | UCN |
| 5-Dihydrocortisol 1 | 1.9236 | 0.64 | 1.71612E-05 | HC |
| caffeic acid | 1.4462 | 3.30E+08 | 0.006376278 | UCN |
| uridine 2 | 1.2823 | 0.29 | 0.000817758 | HC |
| saccharopine 1 | 2.0750 | 1.35E+05 | 6.20166E-05 | UCN |
| canavanine degr prod | 1.7962 | 7.18 | 0.000716324 | UCN |
| o-Hydroxyhippuric acid 1 | 1.7031 | 4.90 | 0.002692949 | UCN |
| Isoxanthopterin | 1.8062 | 23.87 | 6.78102E-08 | UCN |
| Aminooxyacetic acid | 1.9090 | 0.20 | 0.0004583 | HC |
| N-cyclohexylformamide 1 | 1.8967 | 0.68 | 7.60302E-05 | HC |
| Acetol 2 | 1.5697 | 3.74 | 4.55824E-05 | UCN |
| 4-Hydroxybenzoic acid | 1.7531 | 1.61E-06 | 0.000227382 | HC |
| Citraconic acid degr1 | 1.0272 | 0.58 | 7.19859E-06 | HC |
| 2-Deoxyuridine | 1.9662 | 0.05 | 1.09614E-05 | HC |
| HC, healthy control group; UCN, ulcerative colitis with negative *Clostridium difficile* test results; VIP, variable importance for projection. | | | | |
